# Fatigue and symptom-based clusters in post COVID-19 patients: a multicentre, prospective, observational cohort study

**DOI:** 10.1186/s12967-024-04979-1

**Published:** 2024-02-21

**Authors:** Merel E. B. Cornelissen, Lizan D. Bloemsma, Anouk W. Vaes, Nadia Baalbaki, Qichen Deng, Rosanne J. H. C. G. Beijers, Lieke C. E. Noij, Laura Houweling, Somayeh Bazdar, Martijn A. Spruit, Anke H. Maitland-van der Zee, on behalf of the P4O2 Consortium

**Affiliations:** 1 Department of Pulmonary Medicine, Amsterdam UMC, Amsterdam, The Netherlands; 2 Amsterdam Institute for Infection and Immunity, Amsterdam UMC, Amsterdam, The Netherlands; 3 Amsterdam Public Health Research Institute, Amsterdam UMC, Amsterdam, The Netherlands; 4 Department of Research and Development, Ciro, Horn The Netherlands; 5 Department of Respiratory Medicine, Nutrim Institute of Nutrition and Translational Research in Metabolism, Faculty of Health, Medicine and Life Sciences, Maastricht University, Maastricht, The Netherlands; 6 Department of Environmental Epidemiology, Institute for Risk Assessment Sciences (IRAS), Utrecht University, Utrecht, The Netherlands

**Keywords:** Fatigue, Post COVID-19 condition, Long COVID, Post-viral condition, Clusters, ME/CFS, Persistent symptoms

## Abstract

**Background:**

In the Netherlands, the prevalence of post COVID-19 condition is estimated at 12.7% at 90–150 days after SARS-CoV-2 infection. This study aimed to determine the occurrence of fatigue and other symptoms, to assess how many patients meet the Myalgic Encephalomyelitis/Chronic Fatigue Syndrome (ME/CFS) criteria, to identify symptom-based clusters within the P4O2 COVID-19 cohort and to compare these clusters with clusters in a ME/CFS cohort.

**Methods:**

In this multicentre, prospective, observational cohort in the Netherlands, 95 post COVID-19 patients aged 40–65 years were included. Data collection at 3–6 months after infection included demographics, medical history, questionnaires, and a medical examination. Follow-up assessments occurred 9–12 months later, where the same data were collected. Fatigue was determined with the Fatigue Severity Scale (FSS), a score of ≥ 4 means moderate to high fatigue. The frequency and severity of other symptoms and the percentage of patients that meet the ME/CFS criteria were assessed using the DePaul Symptom Questionnaire-2 (DSQ-2). A self-organizing map was used to visualize the clustering of patients based on severity and frequency of 79 symptoms. In a previous study, 337 Dutch ME/CFS patients were clustered based on their symptom scores. The symptom scores of post COVID-19 patients were applied to these clusters to examine whether the same or different clusters were found.

**Results:**

According to the FSS, fatigue was reported by 75.9% of the patients at 3–6 months after infection and by 57.1% of the patients 9–12 months later. Post-exertional malaise, sleep disturbances, pain, and neurocognitive symptoms were also frequently reported, according to the DSQ-2. Over half of the patients (52.7%) met the Fukuda criteria for ME/CFS, while fewer patients met other ME/CFS definitions. Clustering revealed specific symptom patterns and showed that post COVID-19 patients occurred in 11 of the clusters that have been observed in the ME/CFS cohort, where 2 clusters had > 10 patients.

**Conclusions:**

This study shows persistent fatigue and diverse symptomatology in post COVID-19 patients, up to 12–18 months after SARS-CoV-2 infection. Clustering showed that post COVID-19 patients occurred in 11 of the clusters that have been observed in the ME/CFS cohort.

**Supplementary Information:**

The online version contains supplementary material available at 10.1186/s12967-024-04979-1.

## Background

Since the outbreak of severe acute respiratory syndrome coronavirus-2 (SARS-CoV-2) in 2019, more than 700 million cases and almost seven million deaths have been confirmed worldwide [1]. Most patients develop a mild disease with a good prognosis, while over 20% develop a serious or even critical illness [2]. The clinical characteristics and pathogenesis of patients with coronavirus disease 2019 (COVID-19) at the acute phase have been well described, but the long-term consequences are still not fully understood [3].

Previous studies show that some patients do not fully recover after a SARS-CoV-2 infection. Patients who report symptoms that occur 3 months after the acute illness, persist for over 2 months and have no other explanation, have been described as having ‘long-COVID’ or ‘post COVID-19 condition’ [4, 5]. Fatigue seems to be a dominant feature of post COVID-19 condition, along with other symptoms, like cough and dyspnea [5]. In the Netherlands, the prevalence of post COVID-19 condition is estimated at 12.7% at 90–150 days after infection [6].

A recent systematic literature review, mainly including studies examining previously hospitalized COVID-19 patients, has reported the persistence of at least one symptom in 72.5% of the patients, and 49.2% of the patients reported three or more symptoms after ≥ 60 days [7]. Other studies have shown that fatigue, breathlessness and cough are the most common persistent symptoms in post COVID-19 patients [8–11]. Additionally, a study in Arabic countries, who included 965 participants aged ≥ 18 years, found that post COVID-19 patients score significantly higher on fatigue severity, compared to healthy individuals [12]. The number of patients with persistent symptoms seems to decline over time. Indeed, the number of patients with persistent symptoms at day 30 and 180 were 16.9% and 11.7% [9], respectively.

Most studies assessed fatigue with short questionnaires or only studied whether fatigue was present. These types of questionnaires are either not detailed enough or not validated [8–10, 13, 14]. The DePaul Symptom Questionnaire-2 (DSQ-2) is a validated questionnaire to measure myalgic encephalomyelitis/chronic fatigue syndrome (ME/CFS) symptomatology [15]. Perrin et al. [16] suggests that a proportion of COVID-19 patients might develop long-term symptoms similar to ME/CFS. However, only a few case reports of probable or confirmed ME/CFS in post COVID-19 patients have been reported [17] and there still seems to be a lot of variability in immune dysfunction between ME/CFS patients and post COVID-19 patients [18]. ME/CFS is a very complex multi-system disease, often characterized by fatigue that lasts for at least 6 months [19], for which no clear definition is available yet. Instead, there are several criteria, where the most commonly used are The Fukuda CFS Criteria [20], the Canadian ME/CFS Criteria (CCC) [21], the ME International Consensus Criteria (ME-ICC) [22], and the Institute of Medicine Criteria (IOM) [23]. The DSQ-2 may give a good overview of the symptoms in different domains of ME/CFS in patients with post COVID-19 condition.

The aim of this study is therefore to determine the prevalence of fatigue and other symptoms in post COVID-19 patients 3 to 6 months after either hospitalization or a positive SARS-CoV-2 polymerase chain reaction (PCR) test and 9 to 12 months later. The second aim is to determine the number of post COVID-19 patients that meet the ME/CFS criteria according to different definitions. The third aim is to identify clusters in patients with post COVID-19 condition based on the frequency and severity of symptoms and to compare these clusters with clusters observed in a ME/CFS cohort.

## Methods

### Study design and subjects

Precision Medicine for more Oxygen (P4O2) COVID-19 is a multicentre, prospective, observational cohort study. This study was approved by the ethical board of the Amsterdam University Medical Centre (UMC), reference number NL74701.018.20. Details of the study design have been described by Baalbaki et al. [24]. In brief, 95 patients were recruited between May 2021 and September 2022 from post-COVID-19 outpatient clinics in five hospitals in the Netherlands: the Amsterdam UMCs (locations AMC and VUmc), Leiden University Medical Centre, Spaarne Gasthuis in Haarlem, and VieCuri Medical Centre in Venlo.

The post-COVID-19 outpatient clinic was part of standard follow-up care after hospitalization for COVID-19 in the Netherlands. Ex-COVID-19 patients were invited at 3 to 6 months after hospital discharge if they suffered from any persisting symptoms. Additionally, ex-COVID-19 patients who were not hospitalized but suffered from persisting symptoms were referred to the outpatient clinic by their general practitioner at 3 to 6 months after the date of positive PCR or serology test for SARS-CoV-2.

The inclusion criteria for the P4O2 COVID-19 study were: aged 40–65 years, proven ex-COVID-19 (either a positive PCR test, a serology test, and/or a COVID-19 Reporting and Data System (CORADS) score 4/5), the ability to provide informed consent, having access to the internet and understanding the Dutch language. All patients gave their written informed consent. A total of 95 patients were included in the P4O2 COVID-19 study. In the present study, only patients who completed the Fatigue Severity Scale (FSS) at either study visit 1 or 2 or at both study visits were included.

### Study visits

Clinical data about the acute phase of COVID-19 were collected from electronic medical records. The first study visit was planned in parallel to the outpatient clinic visit 3 to 6 months after SARS-CoV-2 infection. During this study visit, general characteristics such as demographics, educational level, smoking, medical history, and medication use were assessed using questionnaires. Additionally, two validated fatigue questionnaires (FSS and DSQ-2) were administered. A second study visit took place 9–12 months later, where the same measurements were performed and the same questionnaires were administered.

### Fatigue severity scale

Patients completed the FSS questionnaire at both study visits. This questionnaire is used to assess the severity of fatigue [25]. Patients rated nine statements on a 7-point Likert scale to assess whether they agree with the statement (1 = strongly disagree, 7 = strongly agree). An average score between 1 and 7 points was calculated, where a higher score means more fatigued. Moderate to high fatigue is defined as having a FSS score ≥ 4 and was used as the cut-off value for further analysis [25].

### DePaul symptom questionnaire-2

When patients scored ≥ 4 on the FSS, they also completed the DSQ-2. This is a self-reported measure of ME/CFS symptomatology, which includes the frequency and severity of 79 symptoms. Both frequency (0 = none of the time, 4 = all of the time) and severity (0 = symptom not present, 4 = very severe) were rated on a 5-point Likert scale. The DSQ-2 has demonstrated to have a strong reliability and validity [15]. First, a composite score was calculated by averaging the frequency and severity score per symptom and multiplying it by 25. This score ranged from 0 to 100 with a higher score indicating a higher symptom burden. Second, a binary “2/2 threshold” was calculated by examining the frequency and severity of each symptom. Patients who reported a score of two or higher for both frequency and severity were considered to have the symptom. Post-exertional malaise (PEM) is defined as the worsening of symptoms following even minor physical or mental exertion and was assessed by examining whether one of the following symptoms met the binary “2/2 threshold”: heavy feeling after starting to exercise, next-day soreness or fatigue after daily activities, mentally or physically tired after minimum exercise, or physically drained after mild activity (Q14, Q15, Q16, Q17 or Q18 of the DSQ-2) [26]. The DSQ-2 can also be used to determine whether patients meet the criteria for the Fukuda case definition, Canadian Consensus Criteria (CCC), International Consensus Criteria for ME (ME-ICC) and/or IOM case definition. A more detailed description on the different criteria can be found in Additional file 1.

### Statistics

Descriptive statistics were reported as mean ± standard deviation (SD) or median (25th–75th percentiles) for continuous data and as frequency (%) for categorical data. Symptom scores were calculated in two ways by using the DSQ-2.

Thereafter, the number of patients that met one or more of the four different definitions for ME/CFS was calculated by using the DSQ-2. Symptoms that were taken into account to calculate these definitions, were e.g. neurological/cognitive problems, unrefreshing sleep, joint pain, sore lymph nodes, muscle aches, PEM, headaches, and a sore throat.

A self-organizing map (SOM) was used to visualize the clustering of patients based on the severity and frequency of 79 symptoms, meaning each patient had 158 features. The SOM method is a non-parametric regression technique that converts multi-dimensional data spaces into lower dimensional abstractions. A SOM generates a non-linear representation of the data distribution and allows to identify homogenous data groups [28]. Missing data were imputed using the Multivariate Imputation by Chained Equations (MICE) package in R studio. Thereafter, the clustering was performed in MATLAB, following its default SOM setting, except for the number of iterations for training the SOM, which was set at 1000 [28]. The default random number generation of MATLAB was used to initialize all competitive units of the SOM, meaning that with the same input and SOM settings, the results are always the same. Vaes et al. [29] clustered 337 ME/CFS patients in the Netherlands into clusters based on their symptom scores. The symptom scores of the post-COVID 19 patients were applied to these clusters to examine whether the same or different clusters were found.

Independent t-tests and chi-square tests were used to test differences in patient characteristics between the two largest clusters.

All analyses were performed with R studio version 4.0.3 (R Foundation for Statistical Computing, Vienna, Austria) and MATLAB (R2022a, MathWorks, MA, USA).

## Results

In total, 91 post COVID-19 patients who either completed the FSS at visit 1, visit 2, or at both visits were included in this study. The mean time between infection and visit 1 is 168 days. The mean age was 53.9 ± 6.1 years and 46 (50.6%) patients were male (Table 1). Of these patients, 81 (89.0%) were hospitalized with a median duration of 8 days and 57 patients (63.3%) reported at least one pre-existing comorbidity. Most reported comorbidities were cardiovascular disease (CVD) (26.4%), asthma (16.5%) and diabetes (14.3%).

**Table 1:** General characteristics of the cohort, n = 91

	Mean ± SD, median (25th–75th percentiles) or n (%)
Age, in years	53.9 ± 6.1
Sex, male	46 (50.6)
Ethnicity	
Caucasian	67/87 (77.0)
African	8/87 (9.2)
Asian	3/87 (3.5)
Latin-American	3/87 (3.5)
Other	6/87 (6.9)
BMI, in kg/m^2^	30.5 ± 5.4
Smoking status	
Current	4 (4.4)
Ex	48 (52.8)
Never	39 (42.9)
Level of education^a^	
Low	33/79 (41.8)
Medium	27/79 (34.2)
High	9/79 (11.4)
At least one comorbidity^b^	57 (63.3)
Comorbidities	
Heart failure	6 (6.6)
Renal failure	5 (5.5)
Diabetes	13 (14.3)
COPD	6 (6.6)
Asthma	15 (16.5)
Cardiovascular disease	
Hospitalized	81 (89.0)
Hospitalization duration, in days	8.0 (4.0, 15.0)
Admitted to ICU	25 (27.5)
Time since infection^c^, in days	167.6 ± 35.9
Acute COVID-19 severity^d^	
Mild	10 (11.0)
Moderate	59 (64.8)
Severe	22 (24.2)

*BMI* Body Mass Index, *COPD* Chronic Obstructive Pulmonary Disease, *ICU* Intensive Care Unit

a. Low = MBO or high school; medium = HBO; high = University bachelor or master

b. Measured comorbidities are COPD, asthma, interstitial lung disease, thrombosis, heart failure, renal failure, hepatic disease, diabetes, cancer, rheumatic disease, CVD and neurologic disease

c. Time between infection and study visit 1

d. According to the WHO definition

### FSS

At the first study visit, 87 patients completed the FSS. The mean ± SD FSS-score of the total population was 5.1 ± 1.6. Of these patients, 66 (75.9%) had a FSS-score of ≥ 4, indicating moderate to high fatigue. A FSS score of ≥ 4 was reported in 31 (68.9%) males and in 35 (83.3%) females. At the second study visit, 76 patients completed the FSS. The mean ± SD FSS-score at the second study visit was 4.4 ± 1.7 and 44 (57.9%) patients had a score ≥ 4. In total, 72 patients completed the FSS at both visits and 38 (52.8%) patients had a score ≥ 4 at both study visits.

### DSQ-2

In total, 61 patients at visit 1 and 39 patients at visit 2 completed the DSQ-2 (Table 2). The DSQ-2 was completed at both visits by 34 patients. Of the patients who scored ≥ 4 on the FSS, 85.0% were also fatigued according to the DSQ-2 at the first study visit, while this percentage was 94.4% at the second study visit. PEM was also a frequently reported symptom, with 72.4% of the patients experiencing PEM at the first visit, and 69.2% reporting it during the second study visit.

**Table 2:** DSQ-2 symptoms at visit 1 and visit 2

	Visit 1 (n = 61)		Visit 2 (n = 39)	
	Composite score^a^ - median (IQR)	Binary score^b^ - frequency (%)	Composite score^a^ - median (IQR)	Binary score^b^ - frequency (%)
Fatigue/Extreme tiredness	62.5 (50.0, 87.5)	51/60 (85.0)	62.5 (50.0, 87.5)	37 (94.9)
Post-exertional malaise				
Dead, heavy feeling after starting exercise	37.5 (6.3, 62.5)	26/60 (43.3)	25.0 (0.0, 59.4)	12 (30.8)
Next-day soreness or fatigue after everyday activities	37.5 (25.0, 62.5)	24 (39.3)	37.5 (25.0, 68.8)	17 (43.6)
Mentally tired after the slightest effort	50.0 (25.0, 75.0)	29/60 (48.3)	25.0 (6.3, 50.0)	12 (30.8)
Physically tired after minimum exercise	50.0 (25.0, 75.0)	31 (50.8)	37.5 (25.0, 71.9)	15 (38.5)
Physically drained or sick after mild activity	37.5 (25.0, 75.0)	25 (41.0)	37.5 (0.0, 59.4)	12 (30.8)
Muscle fatigue after mild physical activity	37.5 (25.0, 75.0)	28/59 (47.5)	25.0 (0.0, 62.5)	15/38 (39.5)
Worsening of symptoms after mild physical activity	25.0 (0.0, 75.0)	21/58 (36.2)	25.0 (0.0, 62.5)	13/38 (34.2)
Worsening of symptoms after mild mental activity	25.0 (0.0, 75.0)	15/58 (25.9)	25.0 (0.0, 50.0)	12/38 (31.6)
Difficulty reading (dyslexia) after mild physical or mental activity	0.0 (0.0, 50.0)	15/59 (25.4)	12.5 (0.0, 46.9)	0/38 (26.3)
Sleep				
Unrefreshing sleep	62.5 (40.6, 75.0)	38/60 (63.3)	50.0 (37.5, 75.0)	23 (59.0)
Need to nap daily	37.5 (6.3, 50.0)	17 (27.9)	37.5 (6.3, 50.0)	11 (28.2)
Problems falling asleep	31.3 (0.0, 59.4)	18/60 (30.0)	25.0 (0.0, 59.4)	12 (30.8)
Problems staying asleep	56.3 (25.0, 75.0)	35/60 (58.3)	50.0 (6.3, 84.4)	20 (51.3)
Waking up early in the morning (e.g. 3 AM)	37.5 (0.0, 75.0)	25/59 (42.4)	25.0 (15.6, 75.0)	16 (41.0)
Sleeping all day and staying awake all night	0.0 (0.0, 25.0)	4 (6.6)	0.0 (0.0, 9.4)	0 (0.0)
Daytime drowsiness	37.5 (25.0, 50.0)	18/59 (30.5)	25.0 (25.0, 50.0)	11/38 (29.0)
Pain				
Pain or aching in muscles	50.0 (25.0, 75.0)	29 (47.5)	50.0 (12.5, 62.5)	20 (51.3)
Joint pain	50.0 (25.0, 75.0)	31 (50.8)	50.0 (25.0, 75.0)	22 (56.4)
Eye pain	0.0 (0.0, 25.0)	6/60 (10.0)	0.0 (0.0, 0.0)	3 (7.7)
Chest pain	0.0 (0.0, 31.3)	7 (11.5)	0.0 (0.0, 25.0)	2 (5.1)
Bloating	25.0 (0.0, 37.5)	11/60 (18.3)	25.0 (0.0, 46.9)	8 (20.5)
Abdomen/stomach pain	0.0 (0.0, 25.0)	6 (9.8)	0.0 (0.0, 25.0)	4 (10.3)
Headaches	37.5 (25.0, 50.0)	18 (29.5)	37.5 (25.0, 50.0)	10 (25.6)
Aching of the eyes or behind the eyes	0.0 (0.0, 0.0)	3/59 (5.1)	0.0 (0.0, 0.0)	1/38 (2.6)
Sensitivity to pain	0.0 (0.0, 25.0)	7/58 (12.1)	0.0 (0.0, 25.0)	6/38 (15.8)
Myofascial pain	0.0 (0.0, 0.0)	7/59 (11.9)	0.0 (0.0, 0.0)	4/38 (10.5)
Neurocognitive				
Muscle twitches	0.0 (0.0, 25.0)	8/60 (13.3)	0.0 (0.0, 25.0)	5/38 (13.2)
Muscle weakness	25.0 (0.0, 50.0)	16 (26.2)	25.0 (0.0, 37.5)	8 (20.5)
Sensitivity to noise	37.5 (0.0, 75.0)	24 (39.3)	37.5 (0.0, 50.0)	13 (33.3)
Sensitivity to bright lights	12.5 (0.0, 50.0)	15 (24.6)	12.5 (0.0, 46.9)	9 (23.1)
Problems remembering things	37.5 (25.0, 75.0)	26 (42.6)	37.5 (12.5, 62.5)	17 (43.6)
Difficulty paying attention for a long period of time	50.0 (25.0, 62.5)	30 (49.2)	37.5 (25.0, 75.0)	18 (46.2)
Difficulty finding the right word to say, or expressing thoughts	37.5 (18.8, 50.0)	17 (27.9)	37.5 (0.0, 62.5)	16 (41.0)
Difficulty understanding things	25.0 (0.0, 37.5)	7/60 (11.7)	12.5 (0.0, 37.5)	8 (20.5)
Only able to focus on one thing at a time	50.0 (0.0, 62.5)	25 (41.0)	25.0 (0.0, 62.5)	16 (41.0)
Unable to focus vision	0.0 (0.0, 25.0)	5 (8.2)	0.0 (0.0, 25.0)	5 (12.8)
Unable to focus attention	18.8 (0.0, 37.5)	9/59 (15.3)	0.0 (0.0, 37.5)	9 (23.1)
Loss of depth perception	0.0 (0.0, 25.0)	4 (6.6)	0.0 (0.0, 0.0)	3 (7.7)
Slowness of thought	25.0 (0.0, 50.0)	13/59 (22.0)	25.0 (0.0, 46.9)	10 (25.6)
Absent-mindedness or forgetfulness	37.5 (25.0, 50.0)	20 (32.8)	25.0 (25.0, 62.5)	14/38 (36.8)
Feeling disoriented	0.0 (0.0, 25.0)	7/59 (11.9)	0.0 (0.0, 6.3)	3/37 (8.1)
Slowed speech	0.0 (0.0, 25.0)	6/59 (10.2)	0.0 (0.0, 37.5)	5/38 (13.2)
Poor coordination	0.0 (0.0, 25.0)	8/59 (13.6)	0.0 (0.0, 25.0)	3/38 (7.9)
Autonomic				
Bladder problems	0.0 (0.0, 25.0)	8/60 (13.3)	0.0 (0.0, 0.0)	2/38 (5.3)
Urinary urgency	18.8 (0.0, 37.5)	12/59 (20.3)	25.0 (0.0, 37.5)	5/38 (13.2)
Waking up at night to urinate	50.0 (25.0, 62.5)	24/59 (40.7)	25.0 (25.0, 62.5)	12/38 (31.6)
Irritable bowel problems	0.0 (0.0, 25.0)	8 (13.1)	0.0 (0.0, 25.0)	6/38 (15.8)
Nausea	0.0 (0.0, 25.0)	3 (4.9)	0.0 (0.0, 0.0)	1/38 (2.6)
Feeling unsteady on feet	12.5 (0.0, 25.0)	7 (11.5)	0.0 (0.0, 25.0)	4/38 (10.5)
Shortness of breath or trouble catching breath	50.0 (25.0, 71.9)	32/60 (53.3)	37.5 (25.0, 50.0)	12/38 (31.6)
Dizziness or fainting	25.0 (0.0, 37.5)	6 (9.8)	0.0 (0.0, 25.0)	1/38 (2.6)
Irregular heartbeats	0.0 (0.0, 25.0)	6 (9.8)	0.0 (0.0, 25.0)	4/38 (10.5)
Heart beats quickly after standing	0.0 (0.0, 37.5)	13/59 (22.0)	0.0 (0.0, 25.0)	7/38 (18.4)
Blurred or tunnel vision after standing	0.0 (0.0, 25.0)	5/59 (8.5)	0.0 (0.0, 0.0)	0/38 (0.0)
Graying or blacking out after standing	0.0 (0.0, 12.5)	0/59 (0.0)	0.0 (0.0, 0.0)	0/38 (0.0)
Inability to tolerate an upright position	0.0 (0.0, 0.0)	2/58 (3.5)	0.0 (0.0, 0.0)	2/38 (5.3)
Neuroendocrine				
Losing weight without trying	0.0 (0.0, 0.0)	4 (6.6)	0.0 (0.0, 0.0.)	1/38 (2.6)
Gaining weight without trying	25.0 (0.0, 43.8)	15 (24.6)	0.0 (0.0, 50.0)	9/38 (23.7)
Lack of appetite	0.0 (0.0, 25.0)	7/60 (11.7)	0.0 (0.0, 0.0)	2/38 (5.3)
Sweating hands	0.0 (0.0, 0.0)	4 (6.6)	(0.0, 0.0)	1/38 (2.6)
Night sweats	0.0 (0.0, 50.0)	17/60 (28.3)	0.0 (0.0, 37.5)	9/38 (23.7)
Cold limbs	18.8 (0.0, 37.5)	8/60 (13.3)	0.0 (0.0, 37.5)	6/38 (15.8)
Feeling chills or shivers	0.0 (0.0, 25.0)	2 (3.3)	0.0 (0.0, 0.0)	3/38 (7.9)
Feeling hot or cold for no reason	0.0 (0.0, 25.0)	5/59 (8.5)	0.0 (0.0, 25.0)	3/38 (7.9)
Felling like you have a high temperature	0.0 (0.0, 31.3)	9/60 (15.0)	0.0 (0.0, 25.0)	2/38 (5.3)
Feeling like you have a low temperature	0.0 (0.0, 0.0)	0/60 (0.0)	0.0 (0.0, 0.0)	1/38 (2.6)
Alcohol intolerance	0.0 (0.0, 0.0)	5/60 (8.3)	0.0 (0.0, 0.0)	1/38 (2.6)
Intolerance to extremes of temperature	0.0 (0.0, 25.0)	11/59 (18.6)	(0.0, 25.0)	6/38 (15.8)
Fluctuations in temperature throughout the day	0.0 (0.0, 0.0)	5/59 (8.5)	0.0 (0.0, 6.3)	3/38 (7.9)
Immune				
Sore throat	6.3 (0.0, 25.0)	5/59 (8.5)	0.0 (0.0, 25.0)	1/38 (2.6)
Tender/sore lymph nodes	0.0 (0.0, 0.0)	2/59 (3.4)	0.0 (0.0, 0.0)	0/38 (0.0)
Fever	0.0 (0.0, 12.5)	4/59 (6.8)	0.0 (0.0, 0.0)	0/38 (0.0)
Flu-like symptoms	25.0 (0.0, 25.0)	4/58 (6.9)	25.0 (0.0, 25.0)	3/38 (7.9)
Sensitivity to smells, food, medications, or chemicals	0.0 (0.0, 0.0)	1/58 (1.7)	0.0 (0.0, 25.0)	3/38 (7.9)
Viral infections with prolonged recovery periods	0.0 (0.0, 37.5)	9/58 (15.5)	0.0 (0.0, 0.0)	0/37 (0.0)
Sinus infections	25.0 (0.0, 37.5)	8/59 (13.6)	0.0 (0.0, 25.0)	3/38 (7.9)
Others				
Sensitivity to mold	0.0 (0.0, 12.5)	5/58 (8.6)	0.0 (0.0, 0.0)	5/38 (13.2)
Sensitivity to vibration	0.0 (0.0, 0.0)	3/59 (5.1)	0.0 (0.0, 0.0)	4/38 (10.5)

a. Composite score: burden of the symptom calculated by taking the mean of the frequency and severity, multiplied by 100

b. Binary score: frequency of the symptom in the cohort. Symptom is present when frequency and severity are both scored ≥ 2

Furthermore, the most reported symptoms at visit 1 and visit 2 were, respectively, unrefreshing sleep in 63.3% and 59.0% of the patients, problems staying asleep in 58.3% and 51.3%, shortness of breath in 53.3% and 31.6%, physically tired after minimum exercise in 50.8% and 38.5%, and joint pain in 50.8% and 56.4% (Table 2).

### ME/CFS case definitions

Additional file 2: Fig S1 shows the number of patients that met the different criteria for ME/CFS at study visit 1 and study visit 2. At the first study visit, six (9.8%) patients met all four different case definitions, whilst 21 (34.4%) of the patients met none of the case definitions. At the second study visit, six (15.4%) patients met all four different case definitions, whilst eight (20.5%) of the patients met none of the case definitions.

### Clustering

**Fig. 1:**
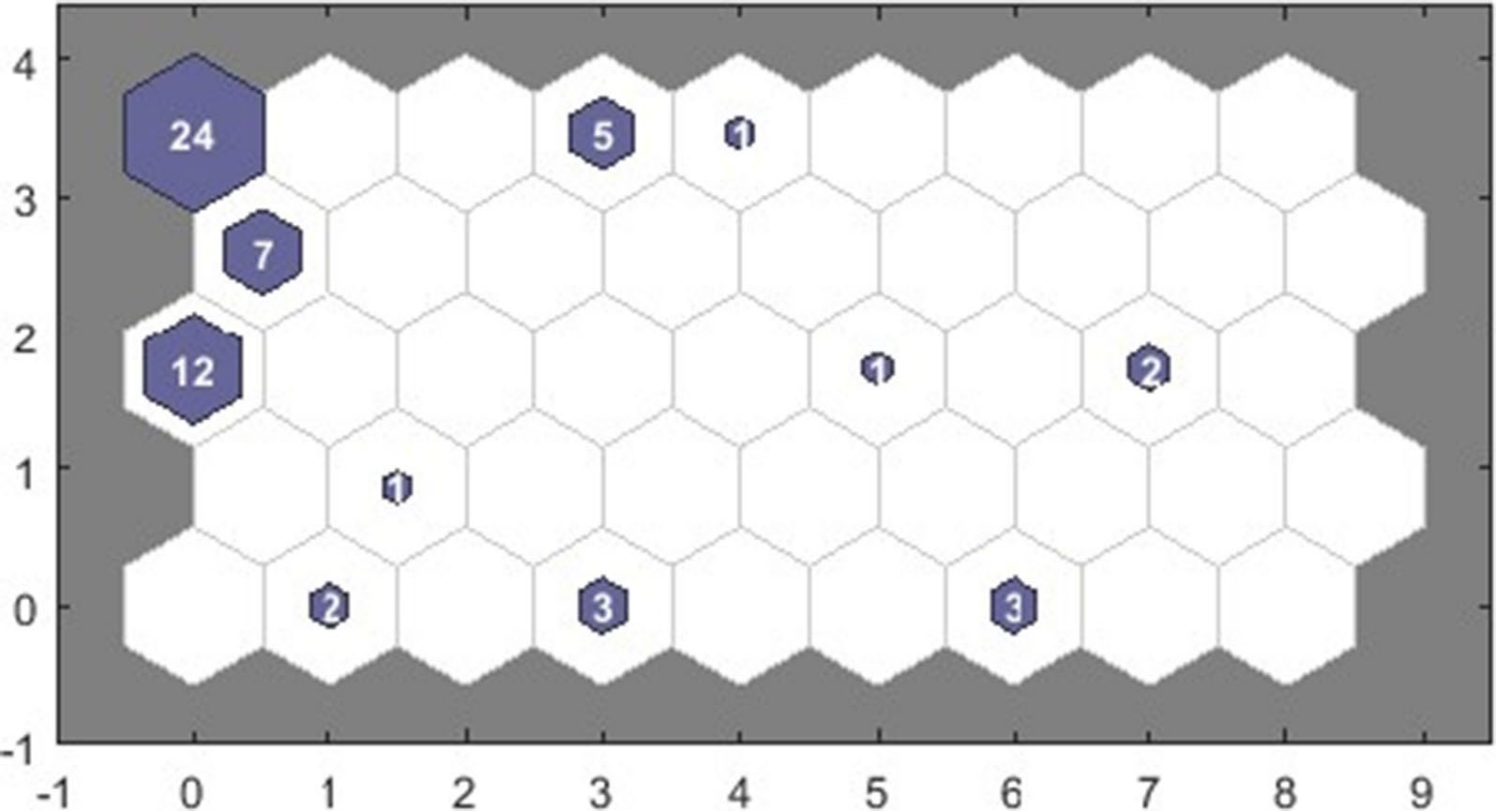
Symptom-based clusters at visit 1 using self-organizing maps. All clusters of patients are displayed in the direction of left to right and bottom to top. Each hexagon represents a cluster, and the number within a hexagon shows the number of patients in the cluster. The x-axis and y-axis indicate the number of clusters, starting from 0. In particular, coordinate (0,0) corresponds to Cluster 1, coordinate (1,0) corresponds to Cluster 2, etc.

Previously, Vaes and colleagues clustered 337 patients with ME/CFS into 45 unique clusters based on their DSQ-2 symptom scores [29]. The scores of the participants of the P4O2 COVID-19 cohort at study visit 1 were applied to these clusters. The post COVID-19 patients occurred in 11 of the ME/CFS clusters (Fig. 1). The five most frequently reported symptoms per cluster with five or more patients can be found in Additional file 3: Table S1.

The characteristics of the two clusters with ≥ 10 patients (clusters 19 and 37) are shown in Table 3. Cluster 19 (n = 12) included more females (75.0%) and patients had on average a slightly higher body mass index (BMI) (33.9 kg/m^2^) compared to the total group that scored ≥ 4 for the FSS (n = 66) where 53.0% were female and the average BMI was 30.9 kg/m^2^. Additionally, cluster 19 contained less patients that worked full time (16.7%) and less patients had a comorbidity (50.0%) compared to the total group with FSS ≥ 4. Cluster 37 (n = 24) contained nearly all current smokers (12.5%), and there were more patients with a comorbidity (70.8%) and more severe acute COVID-19 cases (33.3%). However, all of these characteristics were not statistically significantly different between the two clusters. The mean FSS score in cluster 19 was 6.15 and in cluster 37 was 5.67 (p = 0.04), meaning the patients in cluster 19 were statistically significantly more fatigued.

**Table 3:** Characteristics of patients with FSS ≥ 4 and per cluster that included ≥ 10 patients

	Mean ± SD, median (25th–75th percentiles) or n (%)		
	FSS ≥ 4 (n = 66)	Cluster 19 (n = 12)	Cluster 37 (n = 24)
Age, in years	53.7 ± 6.5	52.7 ± 6.5	54.3 ± 7.3
Gender, male	31 (47.0)	3 (25.0)	13 (54.2)
BMI, in kg/m^2^	30.9 ± 6.0	33.9 ± 5.9	30.0 ± 6.3
Ethnicity			
Caucasian/white	50/63 (79.4)	9 (75.0)	19/22 (86.4)
Other	13/63 (20.6)	3 (24.9)	3/22 (13.5)
Level of education^a^			
Low	24 (36.4)	4 (33.3)	6/23 (26.1)
Medium	30 (45.5)	7 (58.3)	14/23 (60.9)
High	4 (6.1)	1 (8.3)	3/23 (13.0)
Current work status			
On disability	19/63 (30.2)	5 (41.7)	8 (33.3)
Student	2/63 (3.2)	0 (0.0)	1 (4.2)
Homemaker	10/63 (15.9)	4 (33.3)	3 (12.5)
Retired	1/63 (1.6)	0 (0.0)	1 (4.2)
Unemployed	3/63 (4.8)	1 (8.3)	2 (8.3)
Working part-time	18/63 (28.6)	5 (41.7)	2 (8.3)
Working full-time	21/63 (33.3)	2 (16.7)	10 (41.7)
Marital status			
Married/living together	44/59 (74.6)	9 (75.0)	17/23 (73.9)
Living alone	9/59 (15.3)	3 (25.0)	4/23 (17.4)
Widow(er)/divorced	6/59 (10.2)	0 (0.0)	2/23 (8.7)
Smoking status			
Current	4 (6.1)	0 (0.0)	3 (12.5)
Ex-smoker	34 (51.5)	8 (66.7)	11 (45.8)
Never smoker	28 (42.4)	4 (33.3)	10 (41.7)
At least one comorbidity^b^	52 (64.6)	6 (50.0)	17 (70.8)
Comorbidities			
Heart failure	4 (6.1)	0 (0.0)	2 (8.3)
Renal failure	4 (6.1)	0 (0.0)	3 (12.5)
Diabetes	8 (12.1)	0 (0.0)	4 (16.7)
COPD	5 (7.6)	0 (0.0)	2 (8.3)
Asthma	13 (19.7)	2 (16.7)	5 (20.8)
CVD	16 (24.2)	2 (16.7)	6 (25.0)
Hospitalized	56 (84.9)	10 (83.3)	22 (91.7)
Days of hospitalization	7.0 (3.0, 12.0)	7.0 (4.5, 8.0)	8.0 (6.0, 16.0)
ICU	15 (22.7)	1 (8.3)	9 (37.5)
COVID-19 severity^c^			
Mild	10 (15.2)	2 (16.7)	2 (8.3)
Moderate	42 (63.6)	9 (75.0)	14 (58.3)
Severe	14 (21.2)	1 (8.3)	8 (33.3)
FSS score*	5.88 ± 0.78	6.15 ± 0.55	5.67 ± 0.77

*FSS* Fatigue Severity Scale, *BMI* Body Mass Index, *COPD* Chronic Obstructive Pulmonary Disease, *CVD* cardiovascular disease, *ICU* Intensive Care Unit

^*^Differences between the two clusters were statistically significant

a. Low = MBO or high school; medium = HBO; high = University bachelor or master

b. Measured comorbidities are COPD, asthma, interstitial lung disease, thrombosis, heart failure, renal failure, hepatic disease, diabetes, cancer, rheumatic disease, CVD and neurologic disease

c. According to the WHO definition

**Fig. 2:**
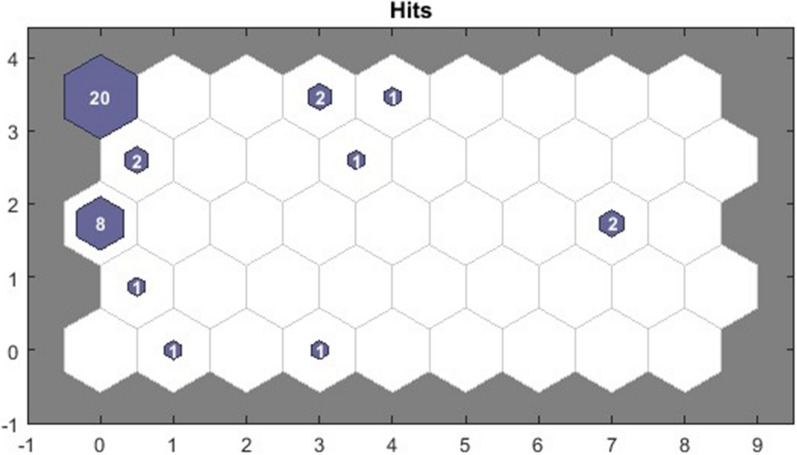
Symptom-based clusters at visit 2 using self-organizing maps. All clusters of patients are displayed in the direction of left to right and bottom to top. Each hexagon represents a cluster, and the number within a hexagon shows the number of patients in the cluster. The x-axis and y-axis indicate the number of clusters, starting from 0. In particular, coordinate (0,0) corresponds to Cluster 1, coordinate (1,0) corresponds to Cluster 2, etc.

When clustering the patients at visit 2, (i.e., 12–18 months after SARS-CoV-2 infection), the same two largest clusters are again observed (clusters 19 and 37) (Fig. 2). However, only nine (26.5%) patients were included in the same cluster at both study visits.

## Discussion

The current study confirms that fatigue and PEM are prominent symptoms in many post COVID-19 patients, which partly recover over time. Furthermore, sleep disturbances, pain, and neurocognitive symptoms were frequently reported. Two-thirds of the post COVID-19 patients met one or more internationally-known ME/CFS definitions. Applying the post COVID-19 data to symptoms-based ME/CFS clusters, we have shown that several ME/CFS clusters do occur in post COVID-19 patients, and that these clusters persisted over time.

The findings of our study are in line with literature. Other studies also report high persistence of fatigue after a SARS-CoV-2 infection. For example, Fortini et al. [11] found fatigue in 42.2% of the cohort at 3–6 months after hospital discharge. This is slightly lower compared to our cohort, however they did not include ICU patients. Tleyjeh et al. showed that 6% of the hospitalized patients reported chronic fatigue syndrome. This is lower than what we found in our cohort (52.7%). This could be due to the use of a different fatigue questionnaire in that study. Another cohort study showed that the rate of post-COVID fatigue decreased over time, only 46.9% of the patients reported this symptom after 6 months compared to 53% at 3 months [10]. Although the percentage of fatigue in our cohort is higher, the decrease over time is in line with our results. When comparing our results with another study conducted in the Netherlands, the percentage of fatigued patients at 3–6 months after acute COVID-19 is more similar [30]. They found 69% to be fatigued, compared to 76% that we found.

PEM is also a highly prevalent symptom in our cohort. Twomey et al. [26] also demonstrated that 94.8% of the post COVID-19 patients experienced PEM, focussing on the presence of one of the first five PEM symptoms of the DSQ-2. In our cohort we observed 72.4% of the patients experiencing PEM during the first study visit. This difference could be due to the selection of participants in this study, since they included patients ≥ 4 weeks post COVID-19 and symptoms could be worse shorter after the acute infection.

At first, it seems reasonable to assume that post COVID-19 patients have similar symptoms as patients with ME/CFS. Nevertheless, one-third of the post COVID-19 patients did not fulfil internationally-known definitions of ME/CFS, which could mean that there are differences between both conditions. However, there are still very few studies that compared both conditions. Jason et al. [31] showed that COVID-19 patients scored higher on e.g. chest pain, shortness of breath and loss of hair, where ME/CFS patients scored higher on neurocognitive symptoms. Next to that, COVID-19 patients showed more improvement over time compared to ME/CFS patients. Then again, several symptoms-based ME/CFS clusters (Vaes et al. [29]) did occur in the post COVID-19 patients, suggesting that several symptom patterns are very similar between both conditions. Therefore, a search for trans-diagnostic predisposing factors of fatigue seems reasonable, which may result in a trans-diagnostic interventions.

Interestingly, the top five features of the post-COVID clusters with five or more patients (Additional file 3: Table S1), show a large variation in the type of symptoms and/or their frequency and severity. These findings again demonstrate the large clinical heterogeneity in daily symptoms, which partly explain the large variation in the challenges patients experience daily.

When comparing the characteristics of the two largest clusters, there were some differences. In cluster 19 there are more females and patients had on average a slightly higher body mass index (BMI) compared to the total group that scored ≥ 4 for the FSS. There were less patients working full time and less patients had a comorbidity. Cluster 37 contained almost all current smokers, and there were more patients with a comorbidity and more severe acute COVID-19 cases.

The biggest ME/CFS cluster (n = 43) was not observed in the P4O2 COVID-19 cohort. This cluster was characterized by high frequency and severity scores for sensitivity to sound, sleeping problems and symptoms after exercise.

### Strengths and limitations

This study has some limitations. First, the sample size is relatively small, which may affect the number and/or size of the post COVID-19 clusters. Larger studies are needed to validate and extend our findings. Specifically, increasing the sample size for clustering analysis could give more reliable comparisons with the ME/CFS cohort. Another limitation of this study is that we did not have a second dataset to validate our findings. This could enhance the external validity of the results and increase the generalizability to other countries and populations. Moreover, we only have two time points where we examined post COVID-19 patients. Since we are interested in the comparison with ME/CFS, a condition that may last for years, it will be of interest to extend the follow-up period in future research to study the progression of symptoms in post COVID-19 patients and make a better comparison with ME/CFS on the long term.

A third limitation is the inconsistency observed in patients’ responses. It was assumed that all patients who completed the DSQ-2 experienced moderate to high fatigue because they all had a FSS score of 4 or higher, yet not all patients reported fatigue on the DSQ-2. This may suggest reporting bias or variations in how patients perceive and express their symptoms and could lead to a degree of outcome misclassification.

A fourth limitation is that we do not know the health status of patients before COVID-19, therefore we do not know whether patients were already fatigued prior to their infection. Also, we did not have a control group with healthy participants. This could have strengthen the results that patients are more fatigue after COVID-19 than before.

Lastly, not all patients who completed the FSS at visit 1, completed the questionnaire at visit 2 and vice versa. This resulted in missing values for the FSS and DSQ-2 at both study visits. However, we did decide to include all data available on the FSS and DSQ-2, otherwise the sample size would be smaller.

An important strength of this study is the use of the DSQ-2, which is an extended, validated questionnaire that assesses not only fatigue but also a broad range of other symptoms. This allows for a more detailed examination of post COVID-19 condition symptoms, beyond just fatigue.

Another strength of this study is the comparison with a cohort of patients suffering from ME/CFS. This provides a better insight into potential overlap and differences between post COVID-19 condition and ME/CFS. This could contribute to a better understanding of post COVID-19 symptomatology and can therefore lead to more targeted treatment of post COVID-19 and ME/CFS.

## Conclusion

This study shows the persistence of fatigue, PEM, and other post COVID-19 symptoms in the P4O2 COVID-19 cohort. The symptom patterns of post COVID-19 patients are similar to a subgroup of patterns known in ME/CFS. These findings highlight the necessity for more research to identify the mechanisms underlying persistent fatigue in post COVID-19 patients in order to prevent it from occurring. These findings highlight the necessity for more research that aims to identify the mechanisms underlying persistent fatigue in post COVID-19. Moreover, at this stage it is hard describe the clinical implications of our findings, since there is still a lot unknown. However, our study does highlight that more research is needed for post COVID-19 condition and ME/CFS, which may lead to better treatment options and an increased quality of life of these patients. For example, intervention studies could be set up to examine whether patients might benefit from certain treatments.

### Supplementary Information

**Additional file 1. **Explanation of the difference case definitions of ME/CFS. There are some similarities and differences between the ME/CFS case definitions, and most ME/CFS patients meet multiple definitions, showing the complexity of ME/CFS. The Fukuda definition identifies a larger and more heterogeneous group of patients compared to the other definitions. Patients fulfilling the CCC have a higher prevalence and severity of symptoms compared to patients fulfilling the Fukuda definition. The CCC and ME-ICC share more similarities, however, patients that meet the ME-ICC have more severe functional impairment and more cognitive problems [27].

**Additional file 2: Figure S1.** The percentage of patients per case definition for ME/CFS at study visit 1 and 2. CCC Canadian Consensus Criteria, ME-ICC International Consensus Criteria for ME, IOM Institute of Medicine Criteria

**Additional file 3: Table S1.** Top 5 features of the clusters with ≥ 5 patients

## Data Availability

The data that support the findings of this study are available from Ortec LogiqCare but restrictions apply to the availability of these data, which were used under license for the current study, and so are not publicly available. Data are however available from the authors upon reasonable request and with permission of Ortec LogiqCare.

## Abbreviations

BMI: Body Mass Index
CCC: Canadian Consensus Criteria
CFS: Chronic Fatigue Syndrome
COPD: Chronic Obstructive Pulmonary Disease
COVID-19: Coronavirus Disease 2019
CVD: Cardiovascular disease
DSQ-2: DePaul Symptom Questionnaire-2
FSS: Fatigue Severity Scale
ICU: Intensive Care Unit
IOM: Institute of Medicine Criteria
ME: Myalgic Encephalomyelitis
ME-ICC: ME International Consensus Criteria
MICE: Multivariate Imputation by Chained Equations
P4O2: Precision Medicine for more Oxygen
PCR: Polymerase Chain Reaction
PEM: Post-exertional malaise
SARS-CoV-2: Severe Acute Respiratory Syndrome Coronavirus 2
SOM: Self-organizing map
UMC: University Medical Centre

## References

[1] World Health Organization (2022). WHO Coronavirus (COVID-19) Dashboard.

[2] Wu Z, McGoogan JM (2020). Characteristics of and important lessons from the coronavirus disease 2019 (COVID-19) outbreak in China: summary of a report of 72314 cases from the Chinese center for disease control and prevention. JAMA.

[3] Wiersinga WJ, Rhodes A, Cheng AC, Peacock SJ, Prescott HC (2020). Pathophysiology, transmission, diagnosis, and treatment of coronavirus disease 2019 (COVID-19): a review. JAMA.

[4] Baig AM (2021). Chronic COVID syndrome: need for an appropriate medical terminology for long-COVID and COVID long-haulers. J Med Virol.

[5] Soriano JB, Murthy S, Marshall JC, Relan P, Diaz JV (2022). A clinical case definition of post-COVID-19 condition by a Delphi consensus. Lancet Infect Dis.

[6] Ballering AV, van Zon SKR, Olde Hartman TC, Rosmalen JGM (2022). Lifelines corona research I. Persistence of somatic symptoms after COVID-19 in the Netherlands: an observational cohort study. Lancet.

[7] Nasserie T, Hittle M, Goodman SN (2021). Assessment of the frequency and variety of persistent symptoms among patients with COVID-19: a systematic review. JAMA Netw Open.

[8] Mandal S, Barnett J, Brill SE, Brown JS, Denneny EK, Hare SS (2021). 'Long-COVID': a cross-sectional study of persisting symptoms, biomarker and imaging abnormalities following hospitalisation for COVID-19. Thorax.

[9] Ong SWX, Fong SW, Young BE, Chan YH, Lee B, Amrun SN (2021). Persistent symptoms and association with inflammatory cytokine signatures in recovered coronavirus disease 2019 patients. Open Forum Infect Dis.

[10] Gonzalez-Hermosillo JA, Martinez-Lopez JP, Carrillo-Lampon SA, Ruiz-Ojeda D, Herrera-Ramirez S, Amezcua-Guerra LM, Martinez-Alvarado MDR (2021). Post-acute COVID-19 symptoms, a potential link with myalgic encephalomyelitis/chronic fatigue syndrome: a 6-month survey in a Mexican cohort. Brain Sci.

[11] Fortini A, Torrigiani A, Sbaragli S, Lo Forte A, Crociani A, Cecchini P (2021). COVID-19: persistence of symptoms and lung alterations after 3–6 months from hospital discharge. Infection.

[12] AlRasheed MM, Al-Aqeel S, Aboheimed GI, AlRasheed NM, Abanmy NO, Alhamid GA (2023). Quality of life, fatigue, and physical symptoms post-COVID-19 condition: a cross-sectional comparative study. Healthcare.

[13] Fernandez-de-Las-Penas C, Palacios-Cena D, Gomez-Mayordomo V, Palacios-Cena M, Rodriguez-Jimenez J, de-la-Llave-Rincon AI (2022). Fatigue and Dyspnoea as main persistent post-COVID-19 symptoms in previously hospitalized patients: related functional limitations and disability. Respiration.

[14] Huang C, Huang L, Wang Y, Li X, Ren L, Gu X (2021). 6-month consequences of COVID-19 in patients discharged from hospital: a cohort study. Lancet.

[15] Bedree H, Sunnquist M, Jason LA (2019). The DePaul symptom questionnaire-2: a validation study. Fatigue.

[16] Perrin R, Riste L, Hann M, Walther A, Mukherjee A, Heald A (2020). Into the looking glass: post-viral syndrome post COVID-19. Med Hypotheses.

[17] Petracek LS, Suskauer SJ, Vickers RF, Patel NR, Violand RL, Swope RL, Rowe PC (2021). Adolescent and young adult ME/CFS after confirmed or probable COVID-19. Front Med.

[18] Peppercorn K, Edgar CD, Kleffmann T, Tate WP (2023). A pilot study on the immune cell proteome of long COVID patients shows changes to physiological pathways similar to those in myalgic encephalomyelitis/chronic fatigue syndrome. Sci Rep.

[19] National Institutes of Health. About ME/CFS? 2022. https://www.nih.gov/mecfs/about-mecfs. Accessed 2 Feb 2024.

[20] Fukuda K, Straus SE, Hickie I, Sharpe MC, Dobbins JG, Komaroff A (1994). The chronic fatigue syndrome: a comprehensive approach to its definition and study. International chronic fatigue syndrome study group. Ann Intern Med.

[21] Carruthers BM, Jain AK, De Meirleir KL, Peterson DL, Klimas NG, Lerner AM (2003). Myalgic encephalomyelitis/chronic fatigue syndrome: clinical working case definition, diagnostic and treatment protocols. J Chron Fat Syndr.

[22] Carruthers BM, van de Sande MI, De Meirleir KL, Klimas NG, Broderick G, Mitchell T (2011). Myalgic encephalomyelitis: international consensus criteria. J Intern Med.

[23] Committee on the Diagnostic Criteria for Myalgic Encephalomyelitis/Chronic Fatigue Syndrome, Board on the Health of Select Population, Institute of Medicine. Beyond Myalgic Encephalomyelitis/Chronic Fatigue Syndrome: Redefining an Illness. Washington (DC): National Academies Press (US). Copyright 2015 by the National Academy of Sciences. All rights reserved. 2015. 25695122

[24] Baalbaki N, Blankestijn JM, Abdel-Aziz MI, de Backer J, Bazdar S, Beekers I (2023). Precision medicine for more oxygen (P4O2)-study design and first results of the long COVID-19 extension. J Pers Med.

[25] meetinstrumenten in de zorg. Fatigue Severity Scale (FSS) 2015. https://meetinstrumentenzorg.nl/instrumenten/fatigue-severity-scale/. Accessed 15 Aug 2023

[26] Twomey R, DeMars J, Franklin K, Culos-Reed SN, Weatherald J, Wrightson JG. Chronic Fatigue and Postexertional Malaise in People Living With Long COVID: An Observational Study. Phys Ther. 2022;102(4). 10.1093/ptj/pzac005 PMC9383197 35079817

[27] Committee on the Diagnostic Criteria for Myalgic Encephalomyelitis/Chronic Fatigue Syndrome; Board on the Health of Select Populations; Institute of Medicine. Beyond Myalgic Encephalomyelitis/Chronic Fatigue Syndrome: Redefining an Illness. Washington (DC): National Academies Press (US); 3, Current Case Definitions and Diagnostic Criteria, Terminology, and Symptom Constructs and Clusters. 2015. Available from: https://www.ncbi.nlm.nih.gov/books/NBK284898/. 25695122

[28] Bullinaria JA. Self organizing maps: fundamentals. Introduction to Neural. 2004;15.

[29] Vaes AW, Van Herck M, Deng Q, Delbressine JM, Jason LA, Spruit MA (2023). Symptom-based clusters in people with ME/CFS: an illustration of clinical variety in a cross-sectional cohort. J Transl Med.

[30] van den Borst B, Peters JB, Brink M, Schoon Y, Bleeker-Rovers CP, Schers H (2021). Comprehensive health assessment 3 months after recovery from acute coronavirus disease 2019 (COVID-19). Clin Infect Dis.

[31] Jason LA, Islam M, Conroy K, Cotler J, Torres C, Johnson M, Mabie B (2021). COVID-19 symptoms over time: comparing long-haulers to ME/CFS. Fatigue.

