# Supplementary material for: Fatigue and symptom-based clusters in post COVID-19 patients: a multicentre, prospective, observational cohort study
Source: J Transl Med. 2024 Feb 21;22:191. doi: 10.1186/s12967-024-04979-1 (PMC10880228; doi:10.1186/s12967-024-04979-1)
Supplement: Supplementary file 1 — Additional file 1. Explanation of the difference case definitions of ME/CFS. There are some similarities and differences between the ME/CFS case definitions, and most ME/CFS patients meet multiple definitions, showing the complexity of ME/CFS. The Fukuda definition identifies a larger and more heterogeneous group of patients compared to the other definitions. Patients fulfilling the CCC have a higher prevalence and severity of symptoms compared to patients fulfilling the Fukuda definition. The CCC and ME-ICC share more similarities, however, patients that meet the ME-ICC have more severe functional impairment and more cognitive problems [27]. [file 12967_2024_4979_MOESM1_ESM.docx]

# Additional files

**Additional file 1: Explanation of the difference case definitions of ME/CFS**

There are some similarities and differences between these definitions, and most ME/CFS patients meet multiple definitions, showing the complexity of ME/CFS. The Fukuda definition identifies a larger and more heterogeneous group of patients compared to the other definitions. Patients fulfilling the CCC have a higher prevalence and severity of symptoms compared to patients fulfilling the Fukuda definition. The CCC and ME-ICC share more similarities, however, patients that meet the ME-ICC have more severe functional impairment and more cognitive problems (27).

**Additional file 2: Figure S1. The percentage of patients per case definition for ME/CFS at study visit 1 and 2**

Abbreviations: CCC = Canadian Consensus Criteria; ME-ICC = International Consensus Criteria for ME; IOM: = Institute of Medicine Criteria

**Additional file 3: Table S1. Top 5 features of the clusters with ≥ 5 patients**

| Group | N | Median score of top 5 features | | | | |
| --- | --- | --- | --- | --- | --- | --- |
| 37 | 24 | Muscle pain | Heavy feeling after exercise | Sensitivity to noise | Heavy feeling after exercise | Difficulty finding words |
|  |  | Severity 1 | Frequency 1 | Severity 0 | Severity 1 | Frequency 1 |
| 19 | 12 | Difficulty finding words | Symptom worsening after mild mental activity | Difficulty understanding things | Difficulty finding words | Muscle twitches |
|  |  | Frequency 3 | Frequency 1 | Severity 2 | Severity 3 | Frequency 1 |
| 28 | 7 | Symptom worsening after mild mental activity | Nausea | Intolerance to extremes of temperature | Sinus infections | Daytime drowsiness |
|  |  | Severity 1 | Frequency 1 | Severity 1 | Severity 1 | Frequency 1 |
| 40 | 5 | Nausea | Bladder problems | Bladder problems | Nausea | Waking up at night to urinate |
|  |  | Severity 2 | Severity 0 | Frequency 0 | Frequency 1 | Frequency 2 |
