# Supplementary material for: Fatigue and symptom-based clusters in post COVID-19 patients: a multicentre, prospective, observational cohort study
Source: J Transl Med. 2024 Feb 21;22:191. doi: 10.1186/s12967-024-04979-1 (PMC10880228; doi:10.1186/s12967-024-04979-1)
Supplement: Supplementary file 2 — Additional file 2: Figure S1. The percentage of patients per case definition for ME/CFS at study visit 1 and 2. CCC Canadian Consensus Criteria, ME-ICC International Consensus Criteria for ME, IOM Institute of Medicine Criteria [file 12967_2024_4979_MOESM2_ESM.docx]

**Additional file 2: Figure S1. The percentage of patients per case definition for ME/CFS at study visit 1 and 2**

Abbreviations: CCC = Canadian Consensus Criteria; ME-ICC = International Consensus Criteria for ME; IOM: = Institute of Medicine Criteria
